# 非血缘脐血干细胞移植治疗高危/难治儿童急性髓系白血病160例临床分析

**DOI:** 10.3760/cma.j.issn.0253-2727.2021.07.004

**Published:** 2021-07

**Authors:** 二玲 陈, 会兰 刘, 良权 耿, 宝林 汤, 小玉 朱, 雯 姚, 闿迪 宋, 湘 皖, 光宇 孙, 萍 强, 倩 范, 紫薇 周, 昌成 郑, 磊 张, 旭晗 张, 娟 童, 自敏 孙

**Affiliations:** 中国科学技术大学附属第一医院（安徽省立医院）血液科，合肥 230001 Anhui Provincial Hospital Affiliated to University of Science and Technology of China Institute of Hematology, Hefei 230001, China

**Keywords:** 脐血干细胞移植, 儿童, 急性髓系白血病, Cord blood hematopoietic stem cell transplantation, Children, Acute myeloid leukemia

## Abstract

**目的:**

探讨单份非血缘脐血干细胞移植（UCBT）治疗高危/难治儿童急性髓系白血病（AML）的疗效。

**方法:**

对2008年6月至2018年12月期间接受UCBT的160例高危/难治AML（不含急性早幼粒细胞白血病）患儿进行回顾性分析。全部病例均采用清髓性预处理方案，应用环孢素A（CsA）联合短程霉酚酸酯（MMF）预防移植物抗宿主病（GVHD）。

**结果:**

160例AML患儿中男103例、女57例，中位年龄7（1～14）岁，中位体重23（8～77）kg。移植后42 d中性粒细胞累积植入率为95.0％（95％*CI* 90.0％～97.5％），移植后120 d血小板累积植入率为85.5％（95％*CI* 83.3％～93.4％），中性粒细胞、血小板植入中位时间分别为移植后16（11～38）d、35（13～158）d。Ⅱ～Ⅳ、Ⅲ～Ⅳ度急性GVHD发生率分别为37.3％（95％*CI* 29.3％～45.2％）、27.3％（95％*CI* 20.0％～35.0％），慢性GVHD发生率为22.4％（95％*CI* 15.5％～28.7％）。可评估的移植后360 d移植相关死亡率（TRM）为13.1％（95％*CI* 8.4％～18.9％）。移植后5年累积复发率为13.8％（95％*CI* 8.5％～20.3％），无病生存（DFS）率为71.7％（95％*CI* 62.7％～77.8％）、总生存（OS）率为72.2％（95％*CI* 64.1％～78.7％），无GVHD无复发生存（GRFS）率为56.1％（95％*CI* 46.1％～64.9％）。移植前处于第1次完全缓解（CR1）期（95例）、第2次完全缓解（CR2）期（28例）、未缓解患者（37例）的移植后5年累积复发率分别为5.3％（95％*CI* 1.9％～11.1％）、19.9％（95％*CI* 6.9％～37.7％）、30.9％（95％*CI* 14.3％～49.2％）（*P*＝0.001），移植后5年OS率分别为79.9％（95％*CI* 70.3％～86.7％）、71.1％（95％*CI* 50.4％～84.4％）、52.9％（95％*CI* 33.0％～69.3％）（*χ*^2^＝7.552，*P*＝0.020）。

**结论:**

UCBT是治愈高危/难治儿童AML安全、有效的治疗方法。第1次完全缓解期进行UCBT有利于改善预后。

## Abstract (en)

**Objective:**

To retrospectively analyze the clinical outcomes of single unrelated cord blood transplantation（UCBT）in children with high risk and refractory acute myeloid leukemia（AML）.

**Methods:**

Between June 2008 and December 2018, a total of 160 consecutive pediatric patients with AML received single UCBT（excluding acute promyelocytic leukemia）. Myeloablative conditioning（MAC）regimen were applied. All patients received a combination of cyclosporine A（CsA）and mycophenolate mofetil（MMF）for the prophylaxis of graft-versus-host disease（GVHD）.

**Results:**

The cumulative incidence of neutrophil cells engraftment at day +42 and platelet recovery at day +120 was 95.0％（95％ *CI* 90.0％–97.5％）at a median of 16 days after transplantation（range, 11–38 days）and 85.5％（95％ *CI* 83.3％–93.4％）with a median time to recovery of 35 days（range, 13–158）, respectively. Incidence of grades Ⅱ-Ⅳ and Ⅲ-Ⅳ acute GVHD and chronic GVHD were 37.3％（95％ CI 29.3％–45.2％）, 27.3％（95％ *CI* 20.0％–35.0％）and 22.4％（95％ *CI* 15.5％–28.7％）, respectively. The transplant-related mortality（TRM）at 360 day was 13.1％（95％ *CI* 8.4％–18.9％）. The 5-year cumulative incidence of relapse was 13.8％（95％ *CI* 8.5％–20.3％）. The 5-year disease-free survival（DFS）and overall survival（OS）were 71.7％（95％ *CI* 62.7％–77.8％）and 72.2％（95％ *CI* 64.1％–78.7％）, respectively. The 5-year GVHD and relapse free survival（GRFS）was 56.1％（95％ *CI* 46.1％–64.9％）. The 5-year cumulative recurrence rates of CR1, CR2, and NR groups were 5.3％, 19.9％, and 30.9％（*P*＝0.001）, and the 5-year OS rates were 79.9％（95％*CI* 70.3％–86.7％）, 71.1％（95％*CI* 50.4％–84.4％）and 52.9％（95％*CI* 33.0％–69.3％）（*χ*^2^＝7.552, *P*＝0.020）, respectively.

**Conclusion:**

For pediatric patients with high risk and refractory AML, UCBT is a safe and effective treatment option, and it is favorable to improve the survival rate in CR1 stage.

儿童急性髓系白血病（AML）占儿童急性白血病的15％～20％[1]。分层治疗及化疗方案优化极大改善了儿童AML的预后，5年总生存（OS）率可达60％[2]–[3]。但单纯化疗对高危及复发/难治的儿童AML疗效有限[4]。造血干细胞移植技术的进步，特别是非血缘脐血干细胞移植（UCBT）在儿童恶性血液病中的应用为更多患儿带来了治愈机会，而且较低的复发率和慢性GVHD发生率显著改善了患儿的长期生存质量[5]。我们对近年来接受UCBT的160例高危/难治儿童AML（不含急性早幼粒细胞白血病）患者进行回顾性分析，旨在探讨UCBT治疗高危/难治儿童AML的远期疗效。

## 病例与方法

1. 病例选择：本研究纳入2008年6月至2018年12月期间在本中心接受UCBT的160例高危/难治儿童AML患者（不含急性早幼粒细胞白血病）。高危定义如下[6]–[8]：①伴有预后差的染色体核型或分子遗传学标志；②骨髓增生异常综合征（MDS）转AML；③高白细胞计数（WBC≥100×10^9^/L）；④合并中枢神经系统白血病（CNSL）。难治定义如下：①经过标准方案治疗2个疗程无效的初治病例；②达完全缓解（CR）并经巩固强化治疗后12个月内复发；③达CR并经巩固强化治疗12个月后复发但常规化疗无效；④多次（≥2次）复发；⑤持续存在髓外白血病。移植指征参考《儿童恶性血液病脐带血移植专家共识》[8]。所有患儿及监护人均在充分告知病情、治疗方案后签署知情同意书，所有操作均获得中国科学技术大学附属第一医院（安徽省立医院）伦理委员会批准。

2. 脐血选择：根据人类白细胞抗原（HLA）相合程度及冻存前总的有核细胞计数（TNC）、CD34^+^细胞数量选择脐血。单份脐血选择原则[9]–[10]：供-受者间HLA位点低分辨配型（HLA-A、-B、-DRB1）≥4/6相合，TNC≥3.0×10^7^/kg（患者体重）且CD34^+^细胞数≥1.2×10^5^/kg（患者体重）。当HLA配型采用HLAA、-B、-C和-DRB1时，尽量选择HLA≥5/8相合；高危患者如果没有其他选择，也可选择HLA 4/8位点相合的脐血。

3. 预处理及GVHD预防方案：均采用清髓性预处理：①以全身照射（TBI）为主方案：TBI总剂量12 Gy（分4次照射），阿糖胞苷（Ara-C）2 g/m^2^每12 h 1次×2 d，环磷酰胺（Cy）60 mg·kg^−1^·d^−1^ ×2 d。②Bu/Cy/Flu方案：氟达拉滨（Flu）30 mg·m^−2^·d^−1^静脉滴注，−8 d～−5 d；白消安（Bu）根据患儿体重给药：<9 kg者1 mg/kg，9～<16 kg者1.2 mg/kg，16～<23 kg者1.1 mg/kg，23～34 kg者0.95 mg/kg，>34 kg者0.8 mg/kg，每6 h 1次静脉滴注，−7 d～−4 d；Cy 60 mg·kg^−1^·d^−1^静脉滴注，−3 d、−2 d。③Bu/Cy/Ara-C方案：Ara-C 2 g/m^2^，每12 h 1次，−9 d、−8 d；Bu和Cy同上。采用化疗预处理的患者，有脑膜白血病史或脑膜白血病高危因素时，加用卡氮芥250 mg/m^2^。

GVHD的预防均采用环孢素A（CsA）联合短程霉酚酸酯（MMF）[5]。

4. 植入前综合征（PES）的诊断及处理：PES是UCBT相对独特且发生率较高的移植相关并发症，定义为中性粒细胞植入6 d前出现的非感染性发热（≥38.3 °C）、非药物所致的红斑性皮疹、非感染性腹泻、体重较基础体重增加10％[11]。根据PES发生时间以及症状轻重进行分层治疗，加用甲泼尼龙（MP）0.5～2 mg·kg^−1^ ·d^−1^静脉滴注，对MP耐药者加用CD25单抗等联合治疗。

5. 支持及对症处理：预防真菌、病毒、肺孢子菌的方案及肝静脉闭塞症、出血性膀胱炎的防治见文献[5]。自移植后6 d（+6 d）开始给予重组人粒细胞集落刺激因子（G-CSF）5～10 µg·kg^−1^·d^−1^，外周血白细胞计数≥4.0×10^9^/L后继续用药2 d。血制品输注前均经X线照射（25 Gy）。

6. 植入检测及随访：所有患儿均留取移植前和移植后第7、14、21 d外周血标本以及移植后28 d骨髓标本，采用短串联重复序列聚合酶链反应（STR-PCR）方法检测植入早期供、患者嵌合体。移植后21 d白细胞无升高趋势且供者嵌合<90％且呈动态下降趋势，28 d后中性粒细胞绝对计数（ANC）<0.5×10^9^/L且STR-PCR检测供者细胞比例<5％定义为原发性植入失败。中性粒细胞及血小板的植入定义见文献[5]。所有患者移植后前4个月每月检测骨髓象、染色体核型、微小残留病（MRD）、WT1融合基因拷贝数及发病时阳性融合基因，移植后半年至1年期间每3个月检测1次，移植后第2～5年每6个月检测1次。GVHD的诊断和分级采用国内指南[12]及NIH标准[13]。采用查阅门诊/住院病历和电话随访方式获得患者生存状况。

7. 统计学处理：采用SPSS 21.0统计软件分析，生存时间以“中位数（范围）”表示。OS率、无病生存（DFS）率、无GVHD无复发生存（GRFS）率采用Kaplan-Meier生存曲线分析，组间生存比较采用Log-rank检验。单因素分析采用Logistic回归分析。粒细胞及血小板植入率、PES发生率、急性GVHD发生率、移植相关死亡率（TRM）和复发率采用R软件竞争风险模型计算累积发生率。累积发生率的比较应用R软件中的Gray检验。*P*<0.05为差异有统计学意义。

## 结果

1. 患者资料：本研究纳入在本中心接受UCBT的160例儿童AML患者，其中男103例、女57例，中位年龄7（1～14）岁，中位体重23（8～77）kg。移植前疾病状态：第1次CR（CR1）95例（59.4％），第2次CR（CR2）28例（17.5％），未缓解（NR）37例（23.1％）；预处理：TBI/Cy/Ara-C方案9例（5.6％），Bu/Cy/Flu方案121例（75.6％），Bu/Cy/Ara-C方案30例（18.8％）。

2. 脐血特征：HLA配型相合度：6/6全相合20例（12.5％），≥4/6相合140例（87.5％）；ABO血型主次均相合83例（52％），不相合77例（48％）。输入复温后脐血TNC中位数为5.23（1.68～17.27）×10^7^/kg，CD34^+^细胞中位数为2.54（0.51～13.78）×10^5^/kg。

3. 造血重建情况：152例患者获得粒系造血重建。移植后42 d中性粒细胞累积植入率为95.0％（95％*CI* 90.0％～97.5％），移植后120 d血小板累积植入率为85.5％（95％*CI* 83.3％～93.4％），中性粒细胞、血小板植入中位时间分别为移植后16（11～38）d、35（13～158）d。单因素分析结果显示，移植前疾病状态、分子遗传学特征、患者年龄、患者体重、患者性别、HLA相合度、ABO血型相合度对植入均无影响。8例未获造血重建患者中5例行挽救性单倍型造血干细胞移植（haplo-HSCT），2例存活至今。

4. GVHD发生情况：86例患者发生急性GVHD，其中Ⅰ度32例；Ⅱ～Ⅳ度54例，累积发生率为37.3％（95％*CI* 29.3％～45.2％）；Ⅲ～Ⅳ度38例，累积发生率为27.3％（95％ *CI* 20.0％～35.0％）。142例患者移植后生存时间超过100 d，其中34例发生慢性GVHD，累积发生率为22.4％（95％ *CI* 15.5％～28.7％），局限型23例，其中22例以皮疹、皮肤脱屑为主要表现，加用MP、芦可替尼和或甲氨蝶呤（MTX）后症状控制，1例出现口腔黏膜干燥、溃疡，使用免疫抑制剂后好转；广泛型慢性GVHD11例，出现皮疹、腹泻和或气喘，加用MP、芦可替尼、MTX、MMF、他克莫司等药物治疗，9例症状控制，2例因肺慢性GVHD死亡。

5. 并发症：全部152例患者中112例在移植后5～17 d诊断为PES，累积发生率为73.7％（95％*CI* 65.6％～79.8％），其中98例在给予MP 0.5～2.0 mg·kg^−1^·d^−1^治疗后症状得到控制，其余14例迁延进展为皮肤、肠道急性GVHD（其中Ⅳ度肠道急性GVHD 10例）。进展为急性GVHD的14例患者中，7例治疗后好转，7例症状未控制，4例因Ⅳ度肠道aGVHD死亡。

29例（18.1％）患者在移植后2～27 d发生血流感染，病原菌包括大肠杆菌（7例）、草绿色链球菌（6例）、肺炎克雷伯杆菌（5例）、奇异变形杆菌（1例）、表皮葡萄球菌（2例）、类干酪乳杆菌（1例）、缓症链球菌（2例）、革兰阳性球菌（3例）、铜绿假单胞菌（1例），屎肠球菌（1例）。101例（63.1％）患者在移植后15～60 d发生巨细胞病毒（CMV）血症。38例患者检出血CMV-DNA>10^3^拷贝数/L，其中1例经肠镜明确为CMV肠炎，1例经肺泡灌洗明确为CMV肺炎，经更昔洛韦/膦甲酸钠联合静脉注射免疫球蛋白治疗后转阴。

23例（14.4％）患者发生出血性膀胱炎，经碱化尿液、利尿、抗病毒等治疗后痊愈。1例患者发生血栓性微血管病变并最终死亡，2例患者发生噬血细胞综合征并最终死亡，1例患者发生肝静脉闭塞病并因咯血死亡。

6. 生存分析：随访截至2020年11月30日，存活患者中位随访时间为4.0（2.0～12.7）年。移植后180 d、360 d TRM分别为11.9％（95％ *CI* 7.4％～17.4％）、13.1％（95％*CI* 8.4％～18.9％）。其中PES组的360 d TRM明显高于未发生PES组［15.2％（95％ *CI* 9.2％～22.5％）对2.5％（95％ *CI* 0.2％～11.4％），*P*＝0.034］。20例患者复发，中位复发时间为移植后157（30～1569）d，仅有1例发生于移植2年后，3例合并髓外复发，其他为单独骨髓复发。移植后5年累积复发率为13.8％（95％*CI* 8.5％～20.3％），PES组、无PES组的移植后5年累积复发率分别为11.4％（95％ *CI* 5.7％～19.2％）、17.5％（95％*CI* 7.6％～30.8％）（*P*＝0.140）。移植前CR1（95例）、CR2（28例）、NR组（37例）移植后5年累积复发率分别为5.3％（95％*CI* 1.9％～11.1％）、19.9％（95％*CI* 6.9％～37.7％）、30.9％（95％*CI* 14.3％～49.2％）（*P*＝0.001）。

移植后5年OS率为72.2％（95％*CI* 64.1％～78.7％），DFS率为71.7％（95％*CI* 62.7％～77.8％），GRFS率为56.1％（95％*CI* 46.1％～64.9％），生存曲线见图1。移植前CR1（95例）、CR2（28例）、NR组（37例）的移植后5年OS率分别为79.9％（95％*CI* 70.3％～86.7％）、71.1％（95％*CI* 50.4％～84.4％）、52.9％（95％ *CI* 33.0％～69.3％）（*χ*^2^＝7.552，*P*＝0.020），生存曲线见图2。

**图1:**
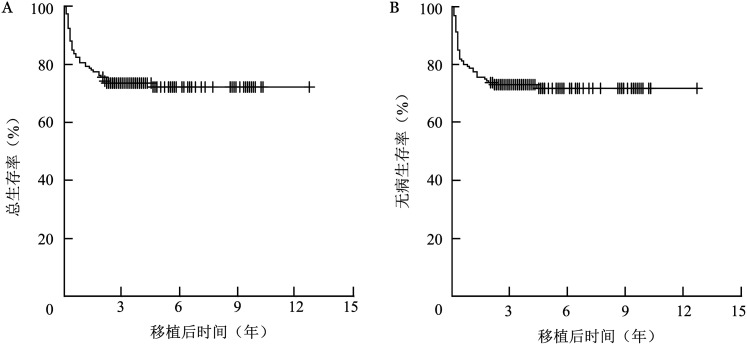
160例高危/难治急性髓系白血病患儿脐血干细胞移植后总生存曲线（A）和无病生存曲线（B）

**图2:**
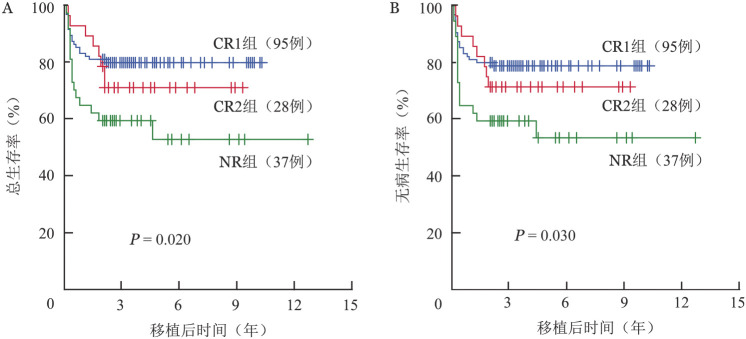
移植前不同疾病状态高危/难治急性髓系白血病患儿脐血干细胞移植后总生存曲线（A）和无病生存曲线（B） CR1：第1次完全缓解；CR2：第2次完全缓解；NR：未缓解

全部160例患者中43例死亡，其中19例（44％）死于复发，24例（55.8％）为移植相关死亡，包括Ⅳ度肠道aGVHD 8例、重症感染7例、多脏器功能衰竭4例、广泛型cGVHD 3例（其中UCBT 2例，haplo-HSCT挽救治疗后1例）、脑出血1例，咯血1例。

## 讨论

本组患儿均采用了不含抗胸腺细胞球蛋白（ATG）的清髓性预处理方案，移植后42 d中性粒细胞累积植入率为95.0％（95％*CI* 90.0％～97.5％）。最近一项纳入331例患者的国内多中心单份UCBT研究[14]显示，TBI/Cy预处理组（200例）粒细胞植入率高于Bu/Cy预处理组（131例）（98.0％对91.6％，*P*<0.001），但Bu/Cy预处理组粒细胞植入时间较TBI/Cy组短（16 d对19 d，*P*<0.001）。本组160例AML患儿资料也得到类似结果（其中126例包含在上述多中心研究中）。

PES是UCBT相对特异的临床表现，不同中心报道的发生率不同[11]。本组病例PES发生率达73.7％（95％*CI* 65.6％～79.8％），其中12.5％的病例迁延进展为急性GVHD。我们既往研究发现，PES和急性GVHD有相关性[15]，大多患者对MP治疗反应良好，但治疗不及时或对MP耐药的患者易迁延进展为急性GVHD，治疗难度大，抗CD25单抗、芦可替尼等二线药物治疗有一定疗效，但仍有部分患者因重度急性GVHD死亡。Isobe等[11]研究发现PES可降低成人AML患者的复发率。本研究PES组、无PES组的移植后5年累积复发率分别为11.4％（95％ *CI* 5.7％～19.2％）、17.5％（95％ *CI* 7.6％～30.8％）（*P*＝0.140），提示PES对儿童AML患者移植后复发率的影响与以往研究不同有待进一步研究。

本组病例均为高危/难治儿童AML患者，UCBT后5年累积复发率仅为13.8％（95％ *CI* 8.5％～20.3％）。复发率低的原因可能与清髓性预处理、弱化GVHD预防方案（仅用CsA及短程MMF且不含ATG），从而缩短移植后免疫重建时间、加快移植物抗白血病作用（GVL）发生有关。单因素分析发现移植前疾病状态是影响复发的高危因素。CR1、CR2、NR组的5年累积复发率分别为5.3％（95％ *CI* 1.9％～11.1％）、19.9％（95％ *CI* 6.9％～37.7％）、30.9％（95％ *CI* 14.3％～49.2％）（*P*＝0.001）。因此，我们认为具有移植指征的高危/难治患儿在CR1期进行UCBT可获得较高的无复发生存率，这与日本的一项多中心研究[16]结果相一致。

本组病例移植后5年DFS、OS率分别为71.7％（95％ *CI* 62.7％～77.8％）、72.2％（95％ *CI* 64.1％～78.7％），GRFS率为56.1％（95％ *CI* 46.1％～64.9％）。CR1组移植后5年OS率高达79.9％。2015年Zheng等[5]比较了UCBT与同胞全相合移植治疗儿童高危/进展期急性白血病的疗效，其中包括了54例AML（UCBT 40例，allo-PBSCT/BMT 14例），UCBT组AML患者的5年OS率为58.1％，5年DFS率为55.7％；allo-PBSCT/BMT组AML患者的5年OS率为55.6％，5年DFS率为32.7％。相较于以往，本组病例的OS和DFS都明显提高，可能与近几年我们对早期并发症（如PES）的识别和处理、治疗GVHD新药的应用以及对HLA配型的认识更加深入等均有一定相关性。

综上，本组病例资料显示，采用不含ATG清髓预处理UCBT是高危/难治AML患儿安全、有效的治疗选择，CR1状态下进行UCBT有利于获得更好的预后。
